# The relationship between social acknowledgment and prolonged grief symptoms: a multiple mediation effect of beliefs about the goodness and controllability of grief-related emotions

**DOI:** 10.1080/20008066.2023.2220633

**Published:** 2023-06-28

**Authors:** Ningning Zhou, Yicheng Wei, Clare Killikelly, Xin Xu, Eva M. Stelzer, Andreas Maercker, Juzhe Xi, Kirsten V. Smith

**Affiliations:** a Shanghai Key Laboratory of Mental Health and Psychological Crisis Intervention, Affiliated Mental Health Center (ECNU), School of Psychology and Cognitive Science, East China Normal University, Shanghai, People’s Republic of China; b Department of Psychiatry, University of British Columbia, Vancouver, Canada; c Department of Psychology, School of Education, Soochow University, Suzhou, People’s Republic of China; d Department of Psychology, University of Arizona, Tucson, AZ, USA; e Centre for Anxiety Disorders and Trauma, Department of Experimental Psychology, University of Oxford, Oxford, UK

**Keywords:** Grief, bereavement, social acknowledgment, emotional beliefs, cross-culture, Duelo, pérdida, reconocimiento social, creencias emocionales, interculturalidad, 哀伤, 丧亲, 社会认可, 情感信念, 跨文化

## Abstract

**Background:** Social acknowledgment is a protective factor for survivors of trauma. However, the role of social acknowledgment in association with prolonged grief symptoms has not yet been established.

**Objectives:** The current study aims to explore the relationship between social acknowledgment and prolonged grief via two beliefs foundational to how people think about grief-related emotions (1) goodness (i.e. whether emotions are desirable, useful, or unwanted and harmful), and (2) controllability (i.e. whether emotions are regulated according to our will or involuntary, arising of their own accord). These effects were explored in two different cultural samples of bereaved people.

**Methods:** One hundred and fifty-four German-speaking and two hundred and sixty-two Chinese bereaved people who lost their loved ones completed questionnaires assessing social acknowledgment, beliefs about the goodness and controllability of grief-related emotions, and prolonged grief symptoms.

**Results:** Correlation analyses showed that social acknowledgment was positively linked with stronger beliefs about the goodness and controllability of grief-related emotions and negatively related to prolonged grief symptoms. Beliefs about the goodness and controllability of grief-related emotions correlated negatively with prolonged grief symptoms. Multiple mediation analyses suggested that beliefs about the controllability and goodness of grief-related emotions mediated the link between social acknowledgment and prolonged grief symptoms. Cultural groups did not moderate the above model.

**Conclusion:** Social acknowledgment may be related to bereavement adjustment consequences via the roles of beliefs about the goodness and controllability of grief-related emotions. These effects seem to be consistent cross-culturally.

## HIGHLIGHTS

- Social acknowledgment correlated positively with stronger beliefs about the goodness and controllability of grief-related emotions and negatively with prolonged grief symptoms.
- Beliefs about the goodness and controllability of grief-related emotions were negatively linked with prolonged grief symptoms.
- Beliefs about the controllability and goodness of grief-related emotions mediated the relationship between social acknowledgment and prolonged grief symptoms. The model presented cross-cultural consistency.

## Abstract (es)

**Antecedentes:** El reconocimiento social es un factor protector para los sobrevivientes de un trauma. Sin embargo, aún no se ha establecido el papel del reconocimiento social en asociación con los síntomas de duelo prolongado.

**Objetivos:** El presente estudio pretende explorar la relación entre el reconocimiento social y el duelo prolongado a través de dos creencias fundamentales en la forma en que las personas piensan sobre las emociones relacionadas con el duelo (1) la bondad (es decir, si las emociones son deseables, útiles, o no deseadas y perjudiciales), y (2) la controlabilidad (es decir, si las emociones se regulan de acuerdo con nuestra voluntad o son involuntarias, surgen por voluntad propia). Estos efectos se exploraron en dos muestras culturales diferentes de personas en duelo.

**Métodos:** Ciento cincuenta y cuatro personas de habla alemana y doscientos sesenta y dos chinos que perdieron a sus seres queridos completaron cuestionarios que evaluaban el reconocimiento social, las creencias sobre la bondad y el control de las emociones relacionadas con el duelo y los síntomas de duelo prolongado.

**Resultados**: Los análisis de correlación mostraron que el reconocimiento social estaba positivamente relacionado con creencias más fuertes sobre la bondad y el control de las emociones relacionadas con el duelo y negativamente relacionado con los síntomas de duelo prolongado. Las creencias sobre la bondad y el control de las emociones relacionadas con el duelo se correlacionaron negativamente con los síntomas de duelo prolongado. Los análisis de mediación múltiple indicaron que las creencias sobre el control y la bondad de las emociones relacionadas con el duelo mediaron el vínculo entre el reconocimiento social y los síntomas de duelo prolongado. Los grupos culturales no moderaron el modelo anterior.

**Conclusiones:** El reconocimiento social puede estar relacionado con las consecuencias de la adaptación al duelo a través de las creencias sobre la bondad y el control de las emociones relacionadas con el duelo. El efecto parece ser consistente transculturalmente.

## Abstract (zh)

**背景：**社会认可对于创伤幸存者是一个保护因素。 然而，社会认可与延长哀伤症状相关的作用尚不确定。

**目的：**当前的研究旨在通过人们如何看待哀伤相关情绪的两种基本信念来探究社会认可与延长哀伤之间的关系 (1) 良好性（即情绪是可取的、有用的，还是不需要的和有害的），以及 (2 ) 可控性（即情绪是根据我们的意愿调节还是非自愿地自行产生）。在两个不同文化的丧亲者样本中考查了这些效应。

**方法：**154 名讲德语的人和 262 名丧亲的中国人完成了问卷调查，评估社会认可、对哀伤相关情绪的良好性和可控性的信念，以及延长哀伤症状。

**结果：**相关性分析表明，社会认可与对哀伤相关情绪的良好性和可控性的强烈信念呈正相关，与延长哀伤症状呈负相关。对哀伤相关情绪的良好性和可控性的信念与延长哀伤症状呈负相关。多重中介分析表明，关于哀伤相关情绪的可控性和良好性的信念中介了社会认可与延长哀伤症状之间的联系。文化群体没有调节上述模式。

**结论：**社会认可可能经由哀伤相关情绪良好性和可控性信念的作用与丧亲调节后果相关。这种效应似乎具有跨文化一致性。

## 1. Introduction

The death of a loved one is considered one of the most stressful and potentially traumatic life events. Although most people recover with time (Sveen et al., 2018), there are still some who continue to experience severe and prolonged grief symptoms and even develop Prolonged Grief Disorder (PGD) (Smith & Ehlers, 2021a). In the International Classification of Diseases for Mortality and Morbidity Statistics (11th Revision) (ICD-11) and the Diagnostic and Statistical Manual of Mental Disorders Fifth Edition Text Revision (DSM-5-TR), PGD is included as a mental disorder (Prigerson et al., 2021; World Health Organization, 2021). Typical symptoms include separation distress, accompanied by accessory symptoms such as guilt, anger, difficulty accepting the death, or a feeling of having lost oneself. In order to meet criteria for a diagnosis these symptoms must persist for at least six months (12 months for DSM-5-TR), generate significant functional impairments, and exceed social, cultural, or religious norms (World Health Organization, 2021). PGD is receiving increasing attention from clinicians and researchers (Killikelly et al., 2020; Kustanti et al., 2021). Prevalence rates following bereavement range from 7–14% worldwide (Kersting et al., 2011; Lundorff et al., 2017; Stroebe et al., 2007), with rates rising to 49% in traumatic loss (Djelantik et al., 2020), signalling an urgent impetus to identify factors that affect the occurrence and development of PGD.

### 1.1. Social acknowledgment and prolonged grief

The experiences of trauma survivors may or may not be acknowledged by the family and community, and this plays a crucial role in their psychological adaptation. For example, aid workers exposed to life-threatening events perceived their traumatic experience as not being acknowledged by people around them (Jones et al., 2006). Some veterans returning from war reported that they experienced low societal appreciation, which predicted more severe post-traumatic symptoms (Forstmeier et al., 2009). Maercker and Miiller (2004) coined the term ‘social acknowledgment’ to describe the trauma survivor’s experience of a positive social response that identifies with their individual state and acknowledges their emotional difficulty. Unlike social support, which only considers the influence of a small social network, social acknowledgment takes into account a larger societal response (Mueller et al., 2009). Social acknowledgment is thought to have three dimensions: recognition as a survivor, general disapproval, and family disapproval. Recognition represents the extent to which the trauma survivor receives care or compassion from friends, neighbours, or colleagues. General disapproval implies the degree to which the survivor’s suffering is acknowledged by society. Family disapproval relates to the perceived reactions (e.g. discomfort) from family members. These dimensions balance positive and negative aspects and take into account family, friends, social environment, and general perceptions of someone in distress (Maercker & Miiller, 2004).

Social acknowledgment is thought to be important in adapting after a significant loss (Wagner et al., 2012), but sometimes bereavement experiences are not acknowledged by others. For example, a qualitative interview study found that bereaved parents benefited when others acknowledged that bereavement was difficult and that the pain would not simply go away (Giannini, 2011). Nevertheless, family members or friends sometimes disparaged the bereaved person for dwelling on the past painful memories, which made them feel worse (Hastings, 2000). What is more, some bereaved individuals experience stigma and become socially ostracized (Pitman et al., 2018). This may lead to disenfranchized grief, meaning that a person’s grief cannot be openly acknowledged, socially validated, or publicly mourned (Albuquerque et al., 2021). Disenfranchized grief is thought to cause difficulties in processing and expressing emotions as well as obtaining social support (Albuquerque et al., 2021).

The extent to which traumatic experiences are acknowledged by the social environment is strongly linked with survivors’ psychological adaptation (Jones et al., 2006; Maercker et al., 2009; Mueller et al., 2009; Wagner et al., 2012). Social acknowledgment was negatively related to post-traumatic stress symptoms among people who experienced workplace violence (Guan et al., 2019). It was also found to be a significant positive predictor of post-traumatic growth for war survivors (Forstmeier et al., 2009). In bereavement research, one study showed that social acknowledgment was a predictor of post-traumatic stress and prolonged grief symptoms among bereaved people witnessing assisted suicide of a loved one (Wagner et al., 2012). Therefore, there may be a negative correlation between social acknowledgment and prolonged grief symptoms. When the experiences of bereaved people are acknowledged and recognized by members of their social network and the communities they inhabit, it is likely that they experience more kindness and may be more accepting of their grief reactions as normal and appropriate (Smith et al., 2020), supporting healthy adaptation. They may exhibit a better adaptation. Conversely, if bereaved people are not allowed to talk about their losses or are evaded by their network, their bereavement experiences are not acknowledged. They may feel their grief reactions are inappropriate or abnormal, and they choose to suppress their emotions, further isolating them from their support network and increasing the risk of prolonged grief.

### 1.2. Beliefs about the goodness and controllability of grief-related emotions as mediators

People may hold various beliefs about the valence of specific emotions (e.g. happiness is good), specific emotion channels (e.g. outwardly expressing anger is bad), or particular contexts (e.g. emotions are detrimental in the workplace) (Ford & Gross, 2018, 2019). Ford and Gross (2019) proposed two beliefs foundational to how people think about emotions: (a) beliefs about whether emotions are good or bad and (b) beliefs about whether emotions are controllable or uncontrollable. In the bereavement context, how grief-related emotions are perceived and how they are linked with prolonged grief symptoms remain unknown. Elaborating on this question can help the researchers and clinicians develop a deeper understanding of grief manifestations and emotional regulation in bereaved people, promoting the design and implementation of tailored intervention strategies for different emotional regulation patterns.

Grief-related emotions may be believed to be beneficial or regarded as helpful for bereaved people. For example, grief-related emotions like yearning or guilt help the bereaved maintain a connection with the deceased and cope with the pain of loss (Oka, 2013). Negative emotions such as sorrow accompanied by grief can increase mutual support and strengthen the social network relationships among survivors (Döveling, 2015). Positive emotions such as joy/happiness in the bereavement context have the potential to enhance interpersonal connections and promote adaptation after loss (Wardecker et al., 2017). Whether grief-related emotions are perceived as good or bad is likely to vary among bereaved people. Some people may perceive them as unhelpful or even harmful. For these people, experiencing grief-related emotions may be interpreted as evidence that they are fragile or out-of-control (Boelen et al., 2003), and expressing them will bring additional emotional distress to other people (Smith et al., 2020). Beliefs about the goodness of emotions are closely related to mental health consequences (Ford & Gross, 2018, 2019). From the social interaction perspective, if individuals believe unpleasant feelings are helpful (Tamir & Ford, 2012), they may be more likely to accept and regulate emotions actively, resulting in more favourable psychological health consequences (Ford & Gross, 2018). When parents believe that their children's anger is valuable, they will respond more positively to their children's negative emotions and promote better psychological states for their children (Halberstadt et al., 2013). In sum, it can be speculated that stronger beliefs about the goodness of grief-related emotions may be linked with a more adaptative post-loss adjustment for bereaved people.

The extent to which grief-related emotions are perceived as controllable by bereaved people is strongly linked with post-loss adaptation. Research has shown that beliefs that emotions are controllable can motivate individuals to regulate their feelings actively and achieve better social adaptation (Tamir et al., 2007). Existing research also demonstrated that beliefs about the controllability over emotions were beneficial to an individual's mental health, such as decreasing depressive and anxiety symptoms (De Castella et al., 2014; Ford et al., 2018). While perceiving high levels of uncontrollability of emotions can lead to experiential avoidance and impede psychological adaptation (Bardeen et al., 2013). Thus, the controllability of grief-related emotions may be negatively related to prolonged grief reactions.

Scholars proposed that emotion-related beliefs may be developed from personal experiences (Ford & Gross, 2018). It can be inferred that when bereaved people perceive more social acknowledgment from their social network relationships and community, they are likely to perceive their grief-related emotions as more acceptable, expected, and less frightening. Conversely, when their bereavement experience is degraded or ignored by their community then they probably experience their grief-related emotions as undesirable or uncontrollable. Previous research has linked negative beliefs about grief-related emotions with an increase in PGD (Boelen & Lensvelt-Mulders, 2005; Smith & Ehlers, 2020; Smith & Ehlers, 2021a, 2021b). Therefore, we hypothesize that this relationship between social acknowledgment and prolonged grief symptoms will be mediated by beliefs about grief-related emotions.

### 1.3. The moderation role of culture

How bereavement experiences are acknowledged by networks surrounding the bereaved and how grief-related emotions are perceived may be relatively influenced by the mourning ceremony, life and death philosophy, religious faith, and individualism-collectivism orientation (Jakoby, 2012). For example, death is taboo in Chinese culture, and people usually avoid talking about it (Cheng et al., 2019). In some cultures, people are not welcome when there is a death in their family (Selin & Rakoff, 2019). These rituals concerning death may make the bereaved feel their bereavement experiences are not acknowledged and prevent them from openly sharing their loss experiences with others (Chan & Chow, 2006). Additionally, some cultures may perceive grief-related emotions as unhelpful or bad. For example, collective culture emphasizes maintaining interpersonal harmony in order to enhance a sense of belonging in a particular group (Shek, 2006), while bereavement has the potential to disrupt social connection if those around the bereaved have not lost loved ones themselves. In such a cultural background, grief-related emotions may threaten significant social relationships for people.

Therefore, culture should be considered when examining the relationships between prolonged grief and social acknowledgment and beliefs about grief-related emotions. We assume that culture plays a moderating role in the relationship between social acknowledgment and prolonged grief via beliefs about grief-related emotions. In the current study, we employed two different cultural groups (i.e. German-speaking vs. Chinese bereaved people) to examine the role of culture in the relationship between social acknowledgment and prolonged grief symptoms via grief-related emotional beliefs. German-speaking bereaved people were mainly recruited from Switzerland, a Western individualistic country, and Chinese bereaved people from China, a typical Eastern collectivistic country. These two cultural groups differ significantly in the individualism-collectivism orientation, religious faith, and life and death philosophy. We proposed the following hypotheses.

**Hypothesis 1.** We expect higher levels of social acknowledgment to be associated with fewer prolonged grief symptoms (Hypothesis 1a). Higher levels of social acknowledgment will be linked with stronger beliefs about the goodness of grief-related emotions (Hypothesis 1b) and stronger beliefs about the controllability of grief-related emotions (Hypothesis 1c). Stronger beliefs about the goodness and controllability of grief-related emotions will correlate with fewer prolonged grief symptoms (Hypothesis 1d and Hypothesis 1e).

**Hypothesis 2.** Beliefs about the goodness of grief-related emotions will mediate the relationship between social acknowledgment and prolonged grief symptoms (Hypothesis 2a). Beliefs about the controllability of grief-related emotions will mediate the relationship between social acknowledgment and prolonged grief symptoms (Hypothesis 2b).

**Hypothesis 3.** Culture will moderate the direct and indirect relationships between social acknowledgment, goodness of grief-related emotions, controllability of grief-related emotions, and prolonged grief symptoms.

## 2. Method

### 2.1. Participants and procedures

Data was part of a large project (i.e. Measurement and Assessment of Prolonged Grief Disorder in China and Switzerland). The project focused on the bereaved people in China and Switzerland. Recruitment criteria included: losing a loved one more than six months ago and less than ten years, age above 18 years, and having no severe mental or physical diseases (e.g. major depression disorder, dementia, suicidal behaviour). The detailed recruitment procedure was described in Killikelly et al. (2020) study. Four hundred and thirty-one participants completed at least 50% of the survey. Fifteen participants were excluded as they did not fill in the questionnaires assessing social acknowledgment, beliefs about grief-related emotions, or prolonged grief symptoms.

Therefore, four hundred and sixteen participants were included in the data analysis (111 males, 301 females, 4 missing values; ages ranging from 18 to 77 years, *M* = 34.12, SD = 13.67). Among them, 154 resided in Switzerland (26 males, 128 females; ages ranging from 18 to 77 years, *M* = 37.0, SD = 16.1), and 262 lived in China (85 males, 173 females; ages ranging from 19 to 70 years, *M* = 32.4, SD = 11.7). Most deaths (80.8%) were due to non-violent causes (i.e. natural death, chronic disease, acute death), and 19.2% percent were caused by accidents, disasters, suicide, or other violent reasons. Detailed demographic and loss-related information is presented in Table 1. The study received ethical approval from the Beijing Normal University, China,^1^ and the University of Zurich, Switzerland (Approval number: 18.8.1).

**Table 1.** Descriptive statistics for sociodemographic and loss-related information in the German-speaking and Chinese bereaved sample.

Variable	German-speaking sample (*n* = 154)		Chinese sample (*n* = 262)		Total sample (*N* = 416)		Difference test
	*M / n*	SD / %	*M / n*	SD / %	*M / n*	SD / %	Two-samples t test or χ2 test
Age (in years) ^a^	37.02	16.10	32.41	11.72	34.11	13.67	*p* = .001
Gender ^a^							*p* < .001
Male	26	16.9%	85	32.4%	111	26.7%	
Female	128	83.1%	173	66.0%	301	72.4%	
Missing			4	1.5%	4	1.0%	
Educational background							*p* < .001
Primary, high, vocational school	79	51.3%	39	14.9%	118	28.4%	
College and university	75	48.7%	223	85.1%	298	71.6%	
Time since loss (in months)	46.68	32.45	49.82	33.63	48.65	33.19	*p* = .352
Kinship							*p* = .001
First degree	82	53.2%	94	35.9%	176	42.3%	
Others	72	46.8%	168	64.1%	240	57.7%	
Cause of death							*p* < .001
Violent	47	30.5%	33	12.6%	80	19.2%	
Non-violent	107	69.5%	229	87.4%	336	80.8%	

Note: The superscript ^a^ represents variables with missing values.

### 2.2. Measures

#### 2.2.1. Social acknowledgment

The Social Acknowledgment Questionnaire (SAQ) measures an individual's perception of being recognized as a trauma survivor in society (Maercker & Miiller, 2004). The original version contains 16 items with three dimensions (i.e. family approval, recognition from other acquaintances, and general approval). We only used the items that assessed social network members’ social acknowledgment and did not include the general disapproval dimension in the current study. Each item ranges from 0 to 3 (0 = not at all; 3 = completely). Therefore, the total scores range from 0 to 30. A higher score indicates a greater sense of being acknowledged. The questionnaire demonstrated moderate internal consistency and reliability for the two cultural groups (*α* = 0.84 for the German-speaking sample, *α* = 0.76 for the Chinese sample).

#### 2.2.2. Beliefs about the goodness of grief-related emotions

By referring to existing studies assessing the beliefs about emotions (Becerra et al., 2020; Halberstadt et al., 2013), we created five items to assess an individual’s beliefs about the goodness of grief-related emotions. Items include ‘It is healthy to grieve’, ‘Grief-related feelings such as sorrow, anger, or worries help people face their loss directly’, ‘Sharing my grief with others gets me support or attention’, and ‘Grief only makes a person feel worse’ (recoded), and ‘There is no benefit to experiencing grief-related feelings’ (recoded). For each item, participants respond with 1–7 (1 = totally disagree, 7 = totally agree). Higher scores represent stronger beliefs about the goodness of grief-related emotions. Cronbach's α coefficient is acceptable in the two samples (*α* = 0.60 for German-speaking; *α* = 0.68 for the Chinese sample).

#### 2.2.3. Beliefs about the controllability of grief-related emotions

Tamir et al.’s (2007) developed the Implicit Theory of Emotion Scale, which consisted of four items, to assess the malleable or controllable nature of emotion. We modified these items to measure beliefs about the fixed versus malleable nature of grief-related emotions. The four items include ‘No matter how hard they try, people cannot really change their grief-related feelings (e.g. sadness, anger about the loss)’ (recoded), ‘Overall, people have very little control over their grief-related feelings’ (recoded), ‘If they want to, people can change the grief-related feelings that they have’, ‘Everyone can learn to control their grief-related feelings’. For each item, participants respond with 1–7 (1 = totally disagree, 7 = totally agree). Higher scores represent more controllability of grief-related emotions. Cronbach's α coefficients were acceptable for the German-speaking sample (*α* = 0.66) but poor for the Chinese sample (*α* = 0.51).

### 2.3. Prolonged grief symptoms

We used the Chinese and German versions of the 13-item International Prolonged Grief Disorder Scale (IPGDS) to measure an individual’s prolonged grief symptoms (Killikelly et al., 2020). The scale is a 5-point Likert scale ranging from 1 (not at all) to 5 (always). Higher scores indicate more severe grief symptoms. Cronbach alpha coefficients were 0.93 in the German-speaking sample and 0.92 in the Chinese sample.

### 2.4. Data analysis

The IBM SPSS 22.0 software package was used for data analyses.

The missing values were less than 1%, and the missing data for each case was less than 10%. Continuous variables were imputed using the average value of each scale, and categorical variables were dealt with using listwise deletion. Descriptive statistics, including mean (*N*) and standard deviation (%), were reported for all the variables. Partial correlation analyses were conducted to investigate the associations among cultural groups (1 = German-speaking bereaved people, 0 = Chinese bereaved people), social acknowledgment, beliefs about the goodness of grief-related emotions, beliefs about the controllability of grief-related emotions, and prolonged grief symptoms. Demographic and loss-related variables including age, gender (0 = male, 1 = female), educational background (0 = primary, middle, high, and vocational school; 1 = college/university), kinship (1 = first-degree relative [parent, child, partner, sibling], 0 = others), and cause of death (1 = violent death [disaster, suicide, accident, etc.], 0 = non-violent death [natural death or disease]) were treated as covariates, as they were found to correlate with the study variables.

The PROCESS macro for SPSS (Model 4) was applied to examine the multiple mediating effects of beliefs about the goodness and controllability of grief-related emotions (Hayes, 2017). The PROCESS macro (Model 59) was applied to examine the moderating effect of the cultural groups on the direct and indirect relationships between social acknowledgment and prolonged grief symptoms. Model 4 and Model 59 were assessed based on the 5000 bootstrap samples (Hayes, 2017). A significant effect was determined by calculating the 95% bias-corrected and accelerated bootstrap confidence intervals (CI). If the 95% CI does not contain 0, the mediating path was deemed to be significant. All study variables were standardized in Model 59.

## 3. Results

### 3.1. Preliminary analyses

Table 2 displayed the mean (N), standard deviation (%), and partial correlation coefficients among cultural groups, social acknowledgment, beliefs about the goodness of grief-related emotions, beliefs about the controllability of grief-related emotions, and prolonged grief symptoms when controlling for age, gender, educational background, kinship and cause of death.

**Table 2.** Mean(N), standardized deviation (%) of study variables and correlation analyses.

Variables	*M* (SD) / *N* (%)	Cultural groups	Social acknowledgment	Beliefs about goodness of grief-related emotions	Beliefs about controllability of grief-related emotions
Cultural groups (1 = German-speaking, 0 = Chinese)	German-speaking: 154 (37.0%) Chinese: 262 (63.0%)	1			
Social acknowledgment	20.27 (5.47)	.02	1		
Beliefs about goodness of grief-related emotions	26.08 (3.22)	−.44***	.29**	1	
Beliefs about controllability of grief-related emotions	13.06 (4.12)	−.11*	.32***	.13**	1
Prolonged grief Symptoms	32.87 (11.49)	.46***	−.45***	−.41***	−.25***

Note: *p < .05, **p < .01, ***p < .001.

Results showed that the cultural group had a negative relationship with beliefs about the goodness of grief-related emotions, had positive relationships with beliefs about the controllability of grief-related emotions and prolonged grief symptoms, but was not linked with social acknowledgment. Social acknowledgment was negatively related to prolonged grief symptoms and positively associated with beliefs about the goodness and controllability of grief-related emotions. Beliefs about the goodness of grief-related emotions were negatively linked with prolonged grief symptoms but not associated with beliefs about the controllability of grief-related emotions. Beliefs about the controllability of grief-related emotions negatively were linked with prolonged grief symptoms.

### 3.2. Testing for the multiple mediation effect

**Figure 1.**
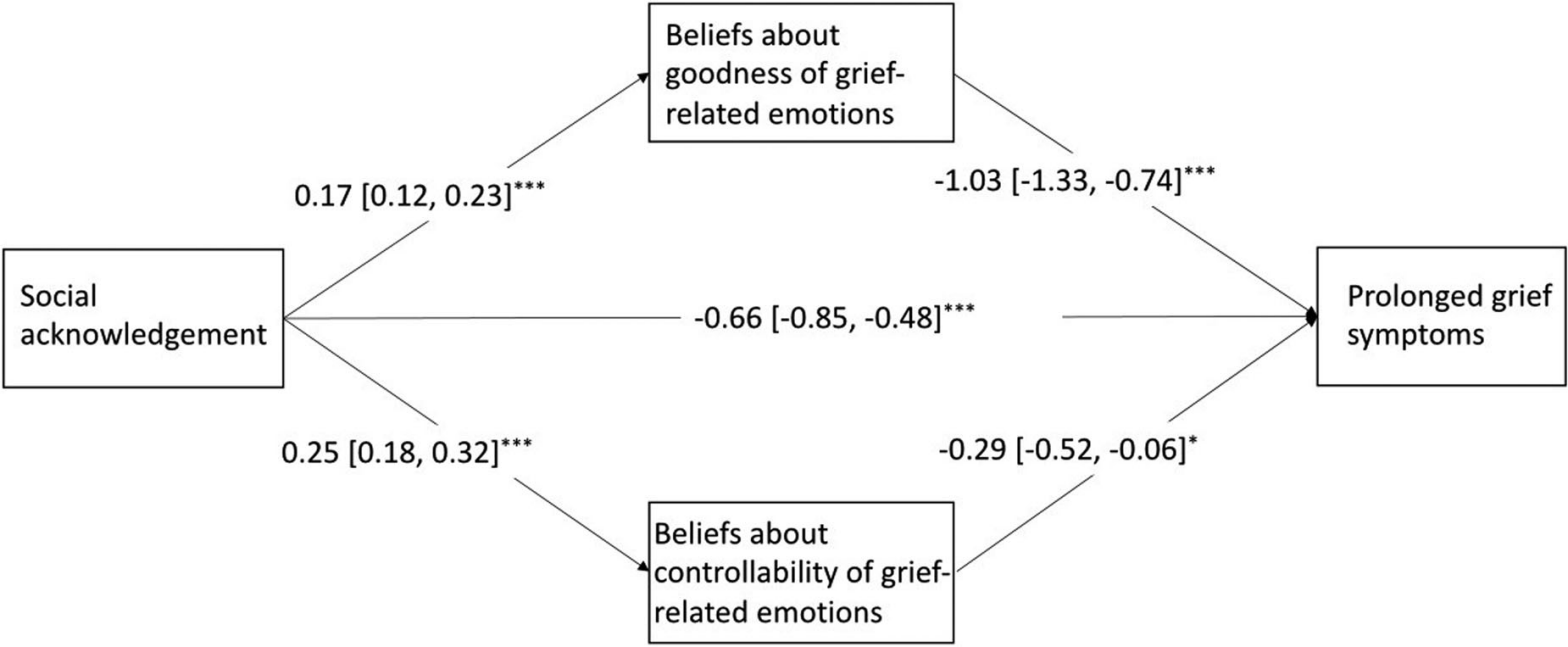
Multiple mediation modeling analysis of the relationship among social acknowledgment, beliefs about the goodness of grief-related emotions, beliefs about the controllability of grief-related emotions, and prolonged grief symptoms. Notes: covariates controlled in the modeling analysis were gender and length of bereavement. *p < .05, **p < .01, ***p < .001.

We used Model 4 of the PROCESS macro to test the multiple mediation effect. Beliefs about the goodness of grief-related emotions partially mediated the relationship between social acknowledgment and prolonged grief symptoms (indirect effect = −0.18, 95% CI = [−0.27, −0.10]). Beliefs about the controllability of grief-related emotions partially mediated the relationship between social acknowledgment and prolonged grief symptoms (indirect effect = −0.07, 95% CI = [−0.14, −0.01]). The mediation effect explained 27.5% of the total effect. The mediation effect of beliefs about the goodness accounted for 19.7% of the total effect, and beliefs about the controllability accounted for 7.8% of the total effect. Results are presented in Figure 1.

### 3.3. Moderated mediation effect analysis

We used Model 59 of the SPSS macro-PROCESS to test the moderated mediation model. Results indicated that cultural groups did not moderate the direct and indirect relationships. Cultural groups did not moderate the relationship between social acknowledgment and prolonged grief symptoms (the interaction term’s coefficient = −0.67, 95% CI = [−0.46, 0.20]). The interaction term’s coefficient between cultural groups and social acknowledgment on beliefs about the goodness of grief-related emotions was −0.02, 95% CI = [−0.12, 0.08], and that between cultural groups and social acknowledgment on beliefs about the controllability of grief-related emotions was −0.03, 95% CI = [−0.17, 0.11]. Cultural groups did not moderate the relationship between beliefs about the goodness of grief-related emotions and prolonged grief symptoms (the interaction term’s coefficient = 0.04, 95% CI = [−0.61, 0.68]) and that between beliefs about the controllability of grief-related emotions and prolonged grief symptoms (the interaction term’s coefficient = −0.14, 95% CI = [−0.56, 0.27]).

## 4. Discussion

This study explores the relationship between social acknowledgment and prolonged grief symptoms via beliefs about the goodness and controllability of grief-related emotions among German-speaking and Chinese bereaved people. The results showed that social acknowledgment was significantly negatively linked with prolonged grief symptoms, supporting Hypothesis 1a. It was significantly positively related to beliefs about the goodness and controllability of grief-related emotions, supporting Hypothesis 1b and 1c. Beliefs about the goodness and controllability of grief-related emotions significantly negatively correlated with prolonged grief symptoms, supporting Hypothesis 1d and 1e. The multiple mediation effects of beliefs about the goodness and controllability of grief-related emotions between social acknowledgment and prolonged grief symptoms were significant, supporting Hypotheses 2a and 2b. The cultural groups did not moderate the direct and indirect relationships of the above multiple mediation model, contradicting Hypothesis 3. However, it should be noted that, the proportion of bereaved people who experienced violent and short-term loss was low, which may bias the results and make subgroup analyses unfeasible. Future research could retest the results in a more diverse sample.

Results supported Hypothesis 1 and established the positive relationships between social acknowledgment and beliefs about the goodness and controllability of grief-related emotions, as well as the negative correlations between prolonged grief symptoms and social acknowledgment, beliefs about the goodness of grief-related emotions, as well as beliefs about the controllability of grief-related emotions. The results indicated that the more social acknowledgment and stronger beliefs about the goodness and controllability of grief-related emotions, the lower their prolonged grief symptoms.

The second hypothesis was supported by showing that social acknowledgment was related to prolonged grief symptoms via beliefs about the goodness and controllability of grief-related emotions. This tentatively suggests that bereaved people who get more social acknowledgment perceive grief-related emotions as good and are able to adapt to their grief more easily, presenting fewer grief symptoms. This supports Hypothesis 2a. Bereaved people who experience more acknowledgment, approval, or acceptance from their social relationships may develop a less negative attitude toward their grief-related feelings (e.g. grief is a natural reaction or grieving is healthy). Rather than criticizing themselves for being immersed in sorrow, they accept or even embrace their grief-related feelings. These people seem to adapt well. Another explanation of the mechanism whereby beliefs about emotions are associated with PGD can be drawn from recent research that demonstrated a causal effect of negative grief-related beliefs, including beliefs about grief-related emotions, on PGD via unhelpful coping strategies (Smith & Ehlers, 2021a). In support of the cognitive model for PTSD (Ehlers & Clark, 2000) and the cognitive behavioural model for complicated grief (Boelen et al., 2006), Smith and Ehlers showed that individuals with more negative beliefs about grief, its emotions, and consequences were more likely to engage in coping strategies aimed avoiding or suppressing their negative thoughts. These unhelpful coping strategies result in the perpetuation of negative beliefs about grief because grievers are not then exposed to situations or circumstances that have the potential to alter their perspective. This vicious cycle is theorized to maintain PGD symptoms. While unhelpful coping strategies were not measured in this study it would be interesting for future research to examine the relationships between social background, the cognitive factors measured here and coping strategies to fully disentangle the route of social acknowledgement to PGD symptoms.

Another perspective that might explain these results is the process of finding benefit in adversity. People who experience misfortunes have a tendency to search for positive influences or find the benefit of a traumatic event (Helgeson et al., 2006), and a supportive social context could foster this (Dunn et al., 2011). It is possible that bereaved people who experience a stronger sense of social acknowledgment more easily identify the positive influence of the loss and even reconstruct meaning from the loss (Neimeyer, 2019). They are likely to develop positive attitudes toward their grief-related feelings. For instance, they realize that grief-related feelings can foster connection with their loved ones, or grief-related emotions have the potential to make them stronger (Calhoun et al., 2013; Joleen et al., 2012). These people can adapt and even grow from the loss.

More social acknowledgment is linked with stronger beliefs in the controllability of grief-related emotions, which is associated with fewer PGD symptoms, supporting Hypothesis 2b. Emotional experience is influenced by social context, and positive experiences can be reinforced in the company of other people (Fischer et al., 2003). Positive social responses from others can encourage and empower the bereaved (Dyregrov, 2004). It is possible that people who experience more positive social responses perceive more autonomy and have a stronger sense of the controllability of their grief-related emotions (Deci & Ryan, 2013). They are likely to adopt adaptative coping strategies to manage their feelings, such as cognitive reappraisal (Ford & Gross, 2018, 2019), and at the same time, decrease the use of nonadaptive coping strategies, such as anxious and depressive avoidance (Boelen et al., 2006). This may mean that they can regulate bereavement-related painful emotions, reframe the meaning of the loss, and adjust to a new life without the deceased.

We did not find a moderating role of culture, contradicting Hypothesis 3. Culture did not moderate the direct and indirect relationships between social acknowledgment and prolonged grief symptoms via beliefs about the goodness or controllability of grief-related emotions. Although there are cultural differences in whether bereaved people perceive grief-related emotions as good or bad or as controllable or uncontrollable, the effects of emotional beliefs on bereavement adjustment seem to be universal cross-culturally. However, further cross-cultural research is needed to verify the current findings.

Notably, social acknowledgment showed no difference between German-speaking and Chinese bereaved people. One possible reason is that other variables, such as social networks, may interfere with the result. For example, if bereaved people have large social networks or perceive satisfactory social support, they are unlikely to feel their bereavement experiences are not acknowledged. Both samples may receive quite a bit of support, and thus the cultural differences in social acknowledgment become insignificant. Future research could investigate this issue by considering the moderation effect of social support or social network size. Another reason may be due to the sample characteristics. Previous studies showed violent or traumatic death tended to be highly influenced by the ‘death taboo’, leading to intense feelings of being not acknowledged (Chapple et al., 2015). In the two cultural groups, most participants lost their loved ones for non-violent reasons. Thus, the grief in this sample may be better acknowledged, and thus the difference of social acknowledgment between cultural groups is not obvious.

### 4.1. Limitations, future directions, and implications

Several limitations of the current study should be noted. Most importantly, these data were cross-sectional, which prevents conclusions regarding causation and the direction of relationship among variables (Cole & Maxwell, 2003; Maxwell & Cole, 2007). For example, it is possible that as a result of an individual’s beliefs about their grief-related emotions being bad and uncontrollable that social network around the griever respond by avoiding their grief in a misguided attempt to help them focus on happier things. Our predictions were based on well-founded theoretical foundations (Boelen et al., 2006), in conjunction with prior findings from longitudinal studies (Boelen & Lensvelt-Mulders, 2005; Smith & Ehlers, 2019; Smith & Ehlers, 2021a, 2021b; Wagner et al., 2012), and as such we believe our cross-sectional mediation may have the potential to shed light on possible causal mechanisms (MacKinnon et al., 2007; Shrout, 2011). However, longitudinal studies are needed to confirm the directionality of the observed effects. Secondly, all the variables are measured by the retrospective method, which may be biased by individuals’ subjective emotional status. Future studies should consider methods with higher ecological validity. For example, experiential sampling or daily diary designs can be used to ascertain how social acknowledgment influences prolonged grief symptoms. Thirdly, the sample is self-selecting from bereavement organizations and platforms and therefore is not representative. Most of the sample had been bereaved for some time and had experienced a non-violent loss. Grievers of non-violent losses may be more likely to seek social acknowledgement from their network than those whose loved one died via violent means. Due to the small numbers of violent losses in our sample we were unable to examine the role of loss type. However, future research with larger samples would be beneficial in elucidating whether a link exists. Fourthly, only Chinese and German-speaking participants were recruited, which cannot fully elaborate on the universality of the model in different cultures. Future studies should recruit bereaved people with various types of loss from diverse cultural backgrounds to further test the model. Finally, it would be interesting to go deeper into the relationships between social acknowledgment and emotional beliefs, as well as the associations between emotional beliefs and prolonged grief symptoms.

The study presents both theoretical and clinical implications. Different from previous studies, which focused primarily on negative cognitions related to grief (Boelen & Lensvelt-Mulders, 2005), the present study identified the beneficial effect of the positive beliefs linked with grief-related feelings. From this point, our findings support and extend the cognitive attachment model proposed by Maccallum and Bryant (2013) and the cognitive behavioural conceptual model proposed by Boelen et al. (2006) by elaborating on the role of adaptive and positive beliefs, appraisals, or cognitions in explaining the remission of prolonged grief symptoms. Additionally, the current study provides evidence for the ‘inside out’ perspective concerning the origin of emotion-related beliefs. That is, personal experiences, such as social acknowledgment after experiencing loss, may contribute to the formation and development of emotion-related beliefs (Ford & Gross, 2018). Furthermore, the current study has the potential to reveal the close ties between the beliefs about the goodness and controllability of emotions and longer-term psychological outcomes after experiencing adverse events such as bereavement (Ford & Gross, 2018, 2019). However, experimental or longitudinal studies are needed to confirm this, especially in those who have experienced sudden and violent losses. Finally, the current research demonstrates the cross-cultural consistency regarding the mediation roles of emotional beliefs between social acknowledgment and prolonged grief symptoms.

The findings provide valuable insights in understanding and intervening in prolonged grief reactions in bereaved people. First, bereaved people seem to experience fewer prolonged grief symptoms when they feel more socially accepted. Relatives or friends can boost social acknowledgment in a number of ways. For example, they can normalize the bereavement experience by increasing acceptance and decreasing discrimination. Alternatively, they can avoid weakening the capability of the bereaved by overprotecting them or accusing them of overreacting. Secondly, working on the beliefs about grief-related emotions is promising to alleviate prolonged grief reactions in bereaved people. The bereaved can be guided to reappraisal and reframe the beliefs of grief-related emotions. For example, psychosocial workers can assist the bereaved in finding the benefits (e.g. love, care, and help they receive) accompanied by their grief-related emotions. In this way, they can recognize the goodness of their grief-related feelings and choose appropriate tactics to regulate their emotions. Practitioners can also help the bereaved be more aware of their resources and ability to increase their controllability on their grief-related feelings and thus better manage these emotions.

## Data Availability

The data of this study are available from the corresponding author. The data are not publicly available due to privacy reasons.

## Note

1 The study has been approved by the ethical committee of Beijing Normal University, China. The ethical committee does not give approval number when approving a study.
